# miRNA Expression Profiles of Mouse Round Spermatids in GRTH/DDX25-Mediated Spermiogenesis: mRNA–miRNA Network Analysis

**DOI:** 10.3390/cells12050756

**Published:** 2023-02-27

**Authors:** Rajakumar Anbazhagan, Raghuveer Kavarthapu, Ryan Dale, Kiersten Campbell, Fabio R. Faucz, Maria L. Dufau

**Affiliations:** 1 Section on Molecular Endocrinology, Division of Developmental Biology, Eunice Kennedy Shriver National Institute of Child Health and Human Development, National Institutes of Health, Bethesda, MD 20892, USA; 2 Bioinformatics and Scientific Programming Core, Eunice Kennedy Shriver National Institute of Child Health and Human Development, National Institutes of Health, Bethesda, MD 20892, USA; 3 Molecular Genomics Core, Eunice Kennedy Shriver National Institute of Child Health and Human Development, National Institutes of Health, Bethesda, MD 20892, USA

**Keywords:** miRNAs, miRNA–mRNA network analysis, GRTH, round spermatids, spermatogenesis, transcriptome analysis

## Abstract

GRTH/DDX25 is a testis-specific DEAD-box family of RNA helicase, which plays an essential role in spermatogenesis and male fertility. There are two forms of GRTH, a 56 kDa non-phosphorylated form and a 61 kDa phosphorylated form (pGRTH). GRTH-KO and GRTH Knock-In (KI) mice with R242H mutation (lack pGRTH) are sterile with a spermatogenic arrest at step 8 of spermiogenesis due to failure of round spermatids (RS) to elongate. We performed mRNA-seq and miRNA-seq analysis on RS of WT, KI, and KO to identify crucial microRNAs (miRNAs) and mRNAs during RS development by establishing a miRNA–mRNA network. We identified increased levels of miRNAs such as miR146, miR122a, miR26a, miR27a, miR150, miR196a, and miR328 that are relevant to spermatogenesis. mRNA–miRNA target analysis on these DE-miRNAs and DE-mRNAs revealed miRNA target genes involved in ubiquitination process (*Ube2k*, *Rnf138*, *Spata3*), RS differentiation, and chromatin remodeling/compaction (*Tnp1/2*, *Prm1/2/3*, *Tssk3/6*), reversible protein phosphorylation (*Pim1*, *Hipk1*, *Csnk1g2*, *Prkcq*, *Ppp2r5a*), and acrosome stability (*Pdzd8*). Post-transcriptional and translational regulation of some of these germ-cell-specific mRNAs by miRNA-regulated translation arrest and/or decay may lead to spermatogenic arrest in KO and KI mice. Our studies demonstrate the importance of pGRTH in the chromatin compaction and remodeling process, which mediates the differentiation of RS into elongated spermatids through miRNA–mRNA interactions.

## 1. Introduction

Spermatogenesis is a highly organized dynamic process whereby male germ cells develop and differentiate serially into mature spermatozoa [1]. This process requires timely coordinated gene expression that is tightly regulated at the transcriptional and post-transcriptional levels. Post-meiotic haploid round spermatids (RS) formed during early spermatogenesis have unique and complex transcriptomes, and precise quality control mechanisms are necessary for the transcribed mRNAs with varied functions [2]. In addition, translational silencing/repression and storage of essential mRNAs occur at specific cytoplasmic sites called chromatoid bodies (CBs) [3,4].

Gonadotropin-regulated testicular RNA helicase (GRTH; DDX25) is a member of the DEAD-box family of RNA helicase first identified in our laboratory, which play essential roles in the completion of spermatogenesis [5,6]. GRTH is expressed exclusively in the Leydig cells and germ cells. It plays several functions as a post-transcriptional regulator of specific genes in germ cells. GRTH knock-out (KO) mice lack sperm and elongating spermatids (ES), making them infertile. These mice exhibit incomplete spermatogenesis due to failure of RS to elongate [5]. In germ cells, the GRTH protein exists in two forms: a 61 kDa phospho form and a 56 kDa non-phospho form. The phospho-GRTH (pGRTH) is found in the cytoplasm and CBs. The non-phosphorylated form is found in the nucleus, in the cytoplasm, and in the CBs. The pGRTH protein in the cytoplasm participates in the shuttling of specific mRNAs in and out of the CBs and becomes associated with polyribosomes for translation. The non-pGRTH protein is involved in the export of specific mRNAs from the nucleus to the cytoplasm of germ cells. Earlier studies from our group revealed a missense mutation (R^242^H) in exon 8 of GRTH gene in non-obstructive azoospermic men, which causes loss of pGRTH protein in COS-1 cells expressing the GRTH (R242H) mutant construct. The 61 kDa phospho-species from the cytoplasm [7] and CBs are absent in GRTH knock-in (KI) transgenic mice (human GRTH gene with R242H mutation), but the non-phospho form from the cytoplasm, nucleus, and CBs is unaffected [4]. Recent studies using GRTH KI and GRTH KO mice revealed an important role of pGRTH in acrosome biogenesis and its structural integrity during spermiogenesis [8]. There is a significant reduction in CB size and complete loss of pGRTH inside CBs, revealing the importance of pGRTH in maintaining the structural integrity of the CB and the associated miRNA pathways [4,7,9]. CBs are dynamic perinuclear organelles that temporarily store mRNAs transported by GRTH from the nucleus to cytoplasm and then finally to the CB [4,10].

During spermatogenesis, the germ cells utilize a large subset of small noncoding regulatory RNAs, such as microRNAs, to control the expression of an array of genes at transcriptional or post-transcriptional levels [1,11,12]. miRNAs are a class of small non-coding RNAs (18–25 nucleotides) that act as endogenous gene regulators and participate in a wide array of biological functions by controlling the stability of mRNAs or promoting target mRNA degradation and inhibition of translation [12,13,14]. miRNAs control posttranscriptional regulation of essential mRNAs to secure the correct timing of translation, which are critical for the final stages of spermiogenesis [10]. Several miRNAs were found to be involved in the regulation of mammalian spermatogenesis and any changes can lead to male infertility [15,16]. miRNAs, such as miR122-5p, can act as potential biomarkers of male infertility [17].

Each miRNA has the capacity to target several mRNAs from many genes at once, thereby tightly regulating gene expression in every organ. Sequence-specific base pairing in the RNA-induced silencing complex with the Argonaute proteins allows miRNAs to identify their target mRNAs (AGO). The Drosha–DGCR8 complex processes primary miRNA transcripts to produce precursor miRNAs, which are where majority of miRNAs originate (pre-miRNAs). These pre-miRNAs are carried to the cytoplasm, where Dicer-dependent or -independent pathways are used to produce mature miRNAs. Germ cells and Sertoli cells have been shown to contain the transcripts for the AGO proteins Drosha and Dicer [11,18]. At the mRNA and protein levels, GRTH controls the expression of numerous microprocessor complex proteins, Drosha, and DGCR8 (which is involved in miRNA synthesis) [11]. In haploid RS, it has been shown that miRNA biogenesis pathway proteins accumulate in the CBs, indicating that the CB and GRTH play a significant role in miRNA-dependent gene regulation [11,19]. The main components of CBs are mRNAs, short RNAs (including piRNA and miRNA), long non-coding RNAs, RNA-binding proteins (including DDX25 and the germ cell marker mouse Vasa homolog [MVH/DDX4]), and other proteins involved in RNA processing [3,4,9]. Owing to the relative importance of RNA regulatory and transport functions of GRTH and its involvement in miRNA biogenesis (at the mRNA and protein levels), it is imperative to study the functions of these components during spermatogenesis.

To understand the precise role of pGRTH in the regulation of germ-cell-specific mRNA and implications of miRNAs during spermiogenesis, RS isolated from germ cells of WT, GRTH-KO, and GRTH-KI mice were analyzed using RNA-seq to compare their transcriptome (mRNA-seq) profiles with miRNA profiles. This study delineates the impact of pGRTH/DDX25 on putative mRNA–miRNA interaction in the regulation of chromatin compaction, remodeling, and ubiquitination processes, which are essential for the progression and completion of spermiogenesis.

## 2. Materials and Methods

### 2.1. Animals

GRTH-KO and GRTH-KI transgenic mice were generated as described previously [5,7]. Briefly, the WT, GRTH-KO, and GRTH KI transgenic mice (10–12 weeks) were genotyped and used for all experiments. All animals were housed in pathogen-free, temperature- and light-controlled conditions (22 °C), with 14 h: 10 h light:dark cycle and ad libitum access to water and food. All animal experiments were performed in accordance with the guidelines established by the National Institute of Child Health and Human Development Animal Care and Use Committee.

### 2.2. Isolation of RS from Mice Seminiferous Tubules

RS were isolated from the testes of GRTH-KO, GRTK-KI, and WT mice (75–85 days old) using a standardized procedure as described previously with minor modifications [20]. Briefly, testes (all genotypes) were decapsulated, seminiferous tubules mildly dispersed and digested using 1 mg/mL collagenase solution (in 1× Krebs buffer; Worthington, Lakewood, NJ, USA) at 37 °C for 3 min to remove Leydig cells. The tubules were washed with Krebs buffer (2 times) at RT and then digested with 0.6 mg/mL trypsin (in 1× Krebs buffer; Sigma-Aldrich, St. Louis, MO, USA) containing DNase I (Thermo Scientific, Waltham, MA, USA) at 34 °C for 15 min (~15 rpm). The obtained germ cell suspension was pre-chilled on ice (7 min) and filtered with a 40 μm filter (Falcon, Corning, NY, USA). The cells were centrifuged (600 g, 5 min, 4 °C) and washed with ice-cold Krebs buffer. The cell pellet was mixed with 3 mL of 0.5% BSA (in 1× Krebs buffer) and filtered again with a 40 μm filter to obtain single-cell suspension of germ cells. The germ cells (in 0.5% BSA) were added onto a BSA gradient (1% to 5% BSA-Krebs Buffer, top to bottom) and allowed to settle for 90 min on ice. Following sedimentation, 1 mL fractions were collected and washed in ice-cold Krebs buffer before cell viability was determined using an automated cell counter (Cell countess, Thermo Scientific). DAPI staining (Thermo Scientific) was used to confirm the purity of the RS cell fractions, which was then examined under a microscope (EVOS M-5000, Thermo Scientific).

### 2.3. mRNA Libraries and Sequencing

RNAeasy Plus micro kit was used to isolate total RNA from six RS samples obtained from the testes of WT, GRTH-KO, and GRTH-KI mice (N = 6; Qiagen, Germantown, MD, USA). RNA quantity and integrity were evaluated in an Agilent Bioanalyzer 2100 system using the RNA Nano 6000 Assay Kit (Agilent Technologies, Santa Clara, CA, USA). Sequencing libraries were prepared using a Takara Pico-RNA-seq kit (Takara) without the polyA selection step. The prepared libraries were quality-checked using a bioanalyzer (Agilent) and used for sequencing using an S1 reagent kit v1.5 (SR100 cycles flow cell (~60 million reads per sample) in a Novaseq 6000 (Illumina, San Diego, CA, USA) platform. The RNA-seq data have been submitted to the Gene Expression Omnibus (GEO; accession number GSE222626).

### 2.4. mRNA-seq Data and Differential Expression of Genes (DEGs)

Quality control was performed on paired-end reads using FastQC (Andrews), RseQC [21], Picard (Broad Institute), and MultiQC [22] both before and after trimming reads with cutadapt v3.4 [23] with arguments -q 20, -A, -a, and –minimum-length 25 (arguments for light quality trimming, adapter removal, and retains only reads >25 bp, respectively). Reads were aligned to the GRCm38 mouse reference genome using the STAR aligner v2.7.8a [24] with arguments --outFilterType BySJout --outFilterMultimapNmax 20 --alignSJoverhangMin 8 --alignSJDBoverhangMin 1 --outFilterMismatchNmax 999 --outFilterMismatchNoverReadLmax 0.04 --alignIntronMin 20 --alignIntronMax 1000000 --alignMatesGapMax 1000000 to match ENCODE standard options for long RNA sequencing. To quantify reads in genes, the GENCODE release 18 annotation was used with featureCounts (in the subread package v2.0.1 [25]) in strand-specific mode (-s2 argument). Differential expression analysis between genotypes was conducted on gene counts using DESeq2 v1.34.0 using the model ~genotype. Genes were identified as differentially expressed if, when using lfcThreshold of 1 to test the null hypothesis that the LFC between conditions is different from 1, they had an adjusted *p*-value < 0.1. Note that the “ashr” shrinkage method [26] was used instead of the default “apeglm” method.

### 2.5. Gene Ontology (GO) and Pathway Analysis

All mRNA identified as differentially expressed in the KO vs. WT and/or KI vs. WT comparisons were further used for functional enrichment analysis, conducted with clusterProfiler v3.18.1 across the GO Biological Processes (BP), Cellular Component (CC), and Molecular Function (MF) databases. Genes were annotated with the three main GO categories (BP, CC, and MF) and were represented separately. A q-value threshold of 0.1 was imposed for the functional enrichment analysis.

### 2.6. Validation of mRNA-seq Data

To validate selected differentially enriched mRNA from RNA-seq transcriptome analyses, real-time quantitative PCR (qRT-PCR) was used. Total RNA was extracted from RS (N = 3) of WT, GRTH-KO, and GRTH-KI mice testes using mRNeasy Micro Kit (Qiagen). Iscript’s first-strand synthesis kit was used to generate cDNA from one microgram of total RNA (Biorad, Hercules, CA, USA). qRT-PCR was carried out on a Quantstudio 3 Fast Real-Time PCR device (Thermo Scientific, CA, USA) with Fast SYBR green and a set of particular gene primers (Supplementary Table S1). All qRT-PCR reactions were performed in triplicates and 18srRNA was used as the reference gene for normalization. The comparative quantification of mRNA was performed using the 2^−ΔΔ^Ct method.

### 2.7. miRNA Libraries and Sequencing

Small RNAs (<200 bp) were purified from isolated RS (>94% purity) obtained from testes of GRTH-KI, GRTH-KO, and WT mice (N = 5) using miRNeasy Micro Kit (Qiagen). RNA quality and quantity were assessed using the RNA Nano 6000 Assay Kit in an Agilent Bioanalyzer 2100 system (Agilent Technologies, CA, USA). Small RNA library construction was carried out using a small RNA-seq Library Kit (Qiagen). The prepared libraries were quality checked using a bioanalyzer (Agilent) and were sequenced using a Novaseq 6000 (Illumina) SP reagent kit v1.5 (100 cycles). The miRNAs were given a general name (e.g., miR26a) without specifying the strand name (e.g., miR26a-5p) for ease of use. All miRNAs identified and discussed in the study are -5p (strand) unless otherwise specified. The miRNA-seq data have been submitted to the Gene Expression Omnibus (accession number GSE222627).

### 2.8. miRNA-seq Data and Differential Abundance of miRNAs

miRNA sequencing analysis was conducted similarly to the described mRNA-seq analysis, with the following modifications. Sequenced reads were obtained in single-ended fastq files. When trimming reads with cutadapt [23], only the -a argument was used to trim QIAseq miRNA 3′ adapters and –minimum-length was reduced to 15 to account for shorter reads following trimming. Reads were aligned to the GRCm38 mouse reference genome using Bowtie2 v2.4.2 [27] with arguments –local, –very-sensitive-local, –k 100 to match suggested parameters for optimized miRNA alignment [28]. Reads were counted in annotated miRNA genes according to the miRBase release 18 annotation, with the appropriate stranded mode (-s1 argument) indicated for featureCounts. When conducting differential expression analysis, two experimental batches were analyzed independently. The KI vs. WT contrast was performed using the first batch and the KO vs. WT contrast was conducted using the second batch. Due to the batch effect, the KI vs. KO contrast could not be confidently executed. No lfcThreshold was imposed for these contrasts.

### 2.9. Validation of miRNA-seq Data

To validate the differentially enriched miRNA obtained from miRNA-seq transcriptome analyses, small RNA prepared from the RS (N = 3) of GRTH-KO, GRTH-KI, and WT were used. Small RNA (<200 bp) was extracted using miRNeasy Micro Kit (Qiagen); quantity and quality were checked prior to cDNA synthesis. Ten nanograms of small RNA (for miRNA quantification) was used to prepare cDNA using the miRCURY LNA RT Kit (Qiagen). qRT-PCR analysis was carried out with set of specific LNA gene probes (Qiagen; Supplementary Table S2) using with miRCURY LNA SYBR^®^ Green (Qiagen) in a Quantstudio 3 Fast Real-Time PCR machine (Thermo Scientific, CA, USA). The following conditions were used: 95 °C for 30 s, followed by 40 cycles of 95 °C for 3s and 60 °C for 30 s. The cycle threshold (Ct) values were normalized to U6 small nuclear RNA as the reference gene, and each experiment was carried out in triplicate. The 2^−ΔΔ^Ct method was used to determine the relative miRNA quantification.

### 2.10. Identification of DE-miRNA–mRNA Target Genes

A stringent set of miRNA–mRNA target pairs were compiled by taking the intersection of three miRNA databases, TargetScan v8.0 [29], TarBase v8 [30], and miRDB v6.0 [31]. This set of predicted miRNA–mRNA target pairs was further filtered to include only miRNAs identified as differentially expressed in this analysis, then subsequently reduced to miRNA–mRNA pairs for which the mRNA target was differentially expressed in the mRNA-seq analysis. This was performed separately for the KO vs. WT and the KI vs. WT. That is, for each of these contrasts, a miRNA–mRNA pair was considered to be supported by this study if all of the following conditions were true: (1) the miRNA–mRNA pair was known or predicted in all three databases; (2) the miRNA was differentially expressed; and (3) the target mRNA was differentially expressed. Note that multiple miRNAs can target one mRNA, and one miRNA can target multiple mRNAs. We did not restrict pairs to be exclusive one-to-one miRNA–mRNA pairs, and we did not restrict pairs to require the opposite direction of miRNA and mRNA so this analysis would include indirect effects.

### 2.11. mRNA–miRNA Interaction Network Analysis

A directed graph was built using the miRNA–mRNA pairs that met the criteria described above, where each pair was further characterized by the following qualities and where “contrast” refers to a single comparison, such as KO vs. WT: (1) miRNA differentially expressed in both contrasts; (2) mRNA differentially expressed in both contrasts; (3) canonical (miRNA and mRNA have opposite log2 fold change sign) or not in a contrast; (4) magnitude of mRNA differential expression in a contrast; (5) identity of miRNA–mRNA targets based on criteria described above. These data were encoded into node and edge attributes of the graph using the networkx Python package and plotted using the matplotlib Python package.

### 2.12. mRNA–miRNA Interaction—Luciferase Assay

COS-1 cells (0.1 × 10^6^ cells/well) were seeded in 12-well plates 24 h before transfection. Cells (70% confluence) were transfected with psiCHECK-2 construct carrying the TP2 coding region alone or together with its 3′ UTR. In addition to DNA constructs, miR122 miRNA mimic (5 nM) alone or miR122 miRNA mimic together with miR122 miRNA inhibitor (20 nM) or negative control (5 nM) were co-transfected using HiPerFect transfection reagent (Qiagen) in a serum-free medium. After incubation (8 h), the media with serum (10%) without antibiotic was changed and further incubated at 37 °C, 5% CO_2_. After 48 h of transfection, the cells were lysed with 1X passive lysis buffer (Promega, WI). Firefly and *Renilla* luciferase activities were measured with the Dual-Luciferase reporter assay system (Promega) using a Glomax navigator microplate luminometer (Promega).

### 2.13. Statistical Analyses

All data were obtained from three or more experiments and the results are presented as mean ± standard error of the mean (SEM). Significance of the differences between the groups was determined by a two-tailed Student t-test using the GraphPad Prism software program (GraphPad Software, Inc., San Diego, CA, USA) and Microsoft Excel (Microsoft). *p* < 0.05 was considered statistically significant.

## 3. Results

### 3.1. Transcriptome Analysis of RS from KO, KI, and WT Mice Reveal Unique RS mRNA Signatures

**Figure 1:**
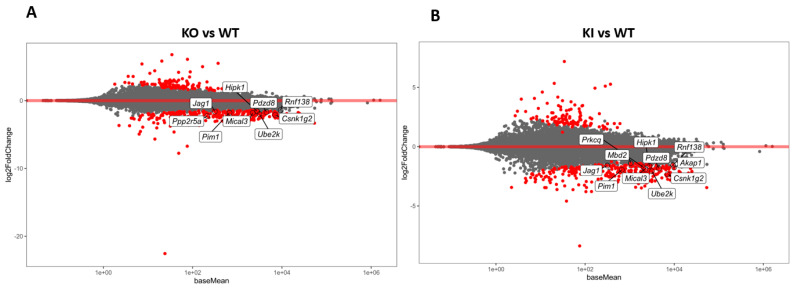
Transcriptome analysis of RS from KO, KI, and WT mice. (**A**) MA plot (log2 fold change vs. average of counts) shows differentially expressed genes (DEGs) from KO and WT (**B**) MA plot (log2 fold change vs. average of counts) shows DEGs from KI and WT groups. The red dots indicate the significantly (*p* < 0.1) upregulated or downregulated genes in each of RS of KO or KI compared to WT.

We initially performed mRNA-seq from the isolated RS of KO, KI, and WT mice (N = 6) to analyze the transcriptome changes in the KO and KI compared to WT. In total, we obtained more than 70 million reads in each RNA-seq library from the RS of KO, KI, and WT mice. Heterogeneity among these individual samples is highlighted in a principal components analysis (PCA) plot (Supplementary Figure S1). WT samples show a clear separation compared to KO and KI, while there is no distinction between KO and KI genotypes. An MA plot (log2 fold change vs. average of counts) was created using the DEGs from KO, KI, and WT groups. Significantly upregulated and downregulated DEGs in each RS of KO or KI compared to WT were shown as red dots (Figure 1A,B). A total of 255 genes downregulated and 114 genes upregulated were identified with a log2 fold change magnitude greater than one fold in the KO group compared to WT. There were 297 genes downregulated and 113 genes upregulated in KI compared to WT. Most enriched mRNAs in KO overlap with KI (Supplementary Figure S3).

### 3.2. Functional Gene Enrichment Analysis of DEGs from RS Transcriptomic Data

**Figure 2:**
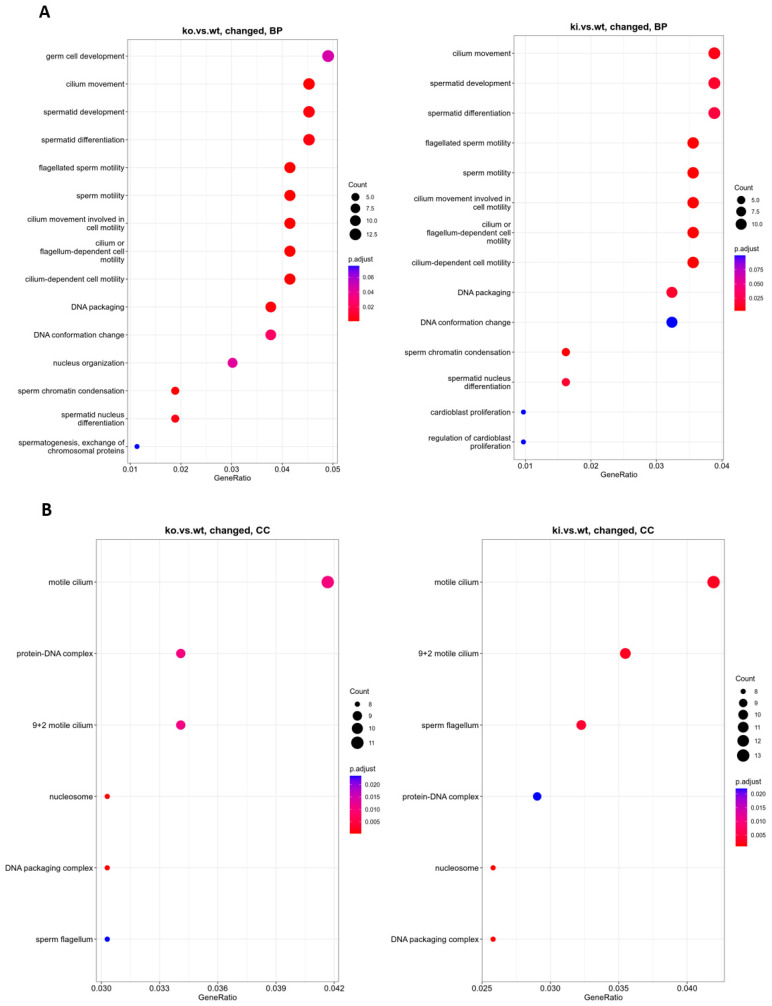
GO functional enrichment analysis on DEGs from RS of KO and KI groups were grouped into different functional categories: biological process (BP), cellular component (CC), and molecular function (MF). (**A**) BP significantly enriched in GO analysis of DEGs (**B**) CC significantly altered in GO analysis of DEGs. The x-axis represents gene ratio = count/set size. The dot size represents the number of genes, and the color of each bar represents the padj-value.

To further analyze and classify the biological function of identified DEGs, we performed functional enrichment analysis using GO in the biological process (BP), cellular component (CC), and molecular function (MF). BP revealed that the genes which play critical roles in germ cell development, spermatid differentiation, and development, such as *Tnp1/2*, *Tssk3/6*, *Prm1/2*, *H2al2a*, *Tbc1d20*, *Fscn3*, *Spem1*, *H1f7*, *Neurl1a*, and *Paqr5,* were altered significantly. DNA packaging and conformation (*Tnp1/2*, *Prm1/2/3*, *H2al2a*, *H2bl1*, *Tssk6*, *H1f7*, *Naa60*), histone exchange (*Prm1/2/3*, *H1f7*), and sperm motility (*Tnp1/2*, *Prm3*, *Smcp*, *Cabs1*, *Gapdhs*, *Spem1*, *Tppp2*, *Efcab1*, *Neurl1a*, *Akap4*) also demonstrated a significant downregulation in KO compared to WT. The BP of KI vs. WT was similar to the KO vs. WT. Changed genes are also enriched in CC categories, including DNA–protein complex, nucleosomes, DNA packaging complex (*Tnp1/2*, *Prm1/2/3*, *H2al2a*, *H2bl1*, *H2ap*), sperm flagella and cilia (*Oaz3*, *Oxct2b*, *Spata18*, *Cabs1*, *Gapdhs*, *Camsap3*, *Atp1b3*, *Tppp2*, *Odf1*, *Efcab1*, *Akap4*) in both KO and KI compared to WT (Figure 2A,B). Thus, differentially expressed genes appear to be related to spermatid differentiation, as would be expected from RS.

### 3.3. Validation of DEGs from mRNA-seq Data Reveals Comparable Transcriptomic Profiles

**Figure 3:**
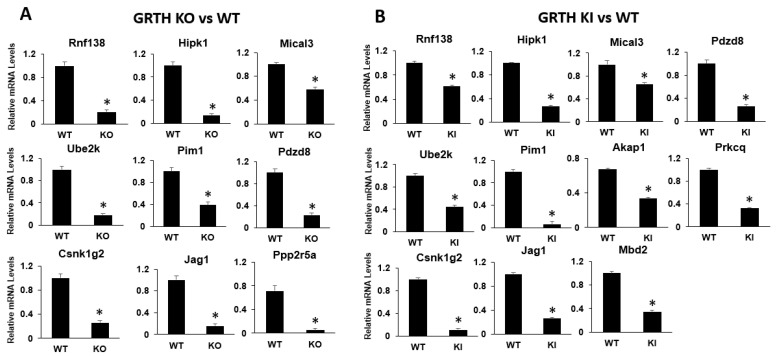
qRT-PCR analysis of selected DEGs of mRNA from RS of KO and KI groups. Bar graphs represent the fold change expression between (**A**) KO vs. WT or (**B**) KI vs. WT groups. Means ± SEM were determined from three independent qRT-PCR experiments with each sample run in triplicates. *p* values were calculated by a two-tailed Student t-test (asterisks indicate *p* < 0.05).

To validate the mRNA-seq transcriptomic data, genes relevant to spermatogenesis and genes which were found to be interacting with identified miRNAs (mRNA–miRNA interaction studies) were selected. Their expression levels were confirmed by qRT-PCR analyses. Genes such as *Rnf138*, *Ube2k*, *Csnk1g2*, *Hipk1*, *Pim1*, *Jag1*, *Mical3*, *Pdzd8*, and *Ppp2r5a* show a significant downregulation (*p* < 0.05) in the RS of KO mice compared to RS of WT mice (Supplementary Table S3). In the RS of KI mice, the transcripts such as *Rnf138*, *Ube2k*, *Csnk1g2*, *Hipk1*, *Pim1*, *Jag1*, *Mical3*, *Pdzd8*, *Akap1*, *Mbd2,* and *Prkcq* downregulated significantly (*p* < 0.05) compared to RS of WT group. *Rnf138* and *Ube2k* are involved in ubiquitination pathways which are critical for the later stages of spermatid development. Genes such as *Jag1* (notch pathway), *Akap1* (regulate cAMP levels), and *Pdzd8* (acrosome stability) regulate spermatogenesis. Other genes such as *Csnk1g2*, *Pim1*, *Prkcq*, *and Ppp2r5a* mediate protein phosphorylation and dephosphorylation. The RNA-seq and GO analysis results suggest altered ubiquitination, phosphorylation, and dephosphorylation events which are comparable with the expression data of qPCR (Figure 3). The list of mRNAs (which are altered and show an interaction with altered miRNA profiles), together with their role in spermatogenesis and general cellular functions, is given in Table 1.

### 3.4. miRNA-seq Profiles of RS from KO, KI, and WT Mice Reveal miRNAs Targeting Spermatid Differentiation Process

**Figure 4:**
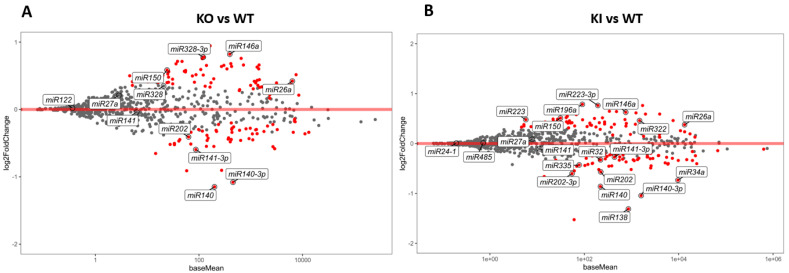
miRNA differential expression in RS from KO, KI, and WT mice. (**A**) MA plot (log2 fold change vs average of counts) shows differentially enriched miRNAs from KO and WT group (**B**) MA plot (log2 fold change vs average of counts) shows DEGs from KI and WT group. The red dots indicate the significantly (*p* < 0.1) upregulated or downregulated miRNAs in each of RS of KO or KI compared to WT.

Since each mRNA is regulated by more than one miRNA and one miRNA regulates more than one mRNA, it is essential to assess the entire RS population of miRNAs. miRNA-seq was performed from the isolated RS of KO, KI, and WT mice (N = 5). A total of around 80 million reads was obtained from each miRNA-seq library made from the RS of KO, KI, and WT mice. miRNA-seq was carried out in two different batches, KO vs. WT and KI vs. WT, and the overall results were compared between all the genotypes. A PCA plot of these samples shows a clear separation of WT compared to KO and KI, while there is no distinction between KO and KI genotypes (Supplementary Figure S2). Several miRNAs, such as miR32, miR184, miR335, miR140, miR141, miR1981, miR202, miR880, and miR669c, were downregulated in both KO and KI mice (Figure 4; Supplementary Table S4). These are involved in the positive regulation of spermatid differentiation, sperm motility, mRNA processing, and decay. The miRNAs miR150, miR196a-2, miR652, miR146, miR10b, miR379, miR122a, miR26a, miR27a, miR127, and miR328 were upregulated in both KO and KI mice (Figure 4; Supplementary Table S4), which negatively regulate the spermatid differentiation. Several enriched miRNAs in KO overlap with KI (Supplementary Figure S4).

### 3.5. Validation of Differential Gene Expression Analysis of miRNAs Using qRT-PCR

**Figure 5:**
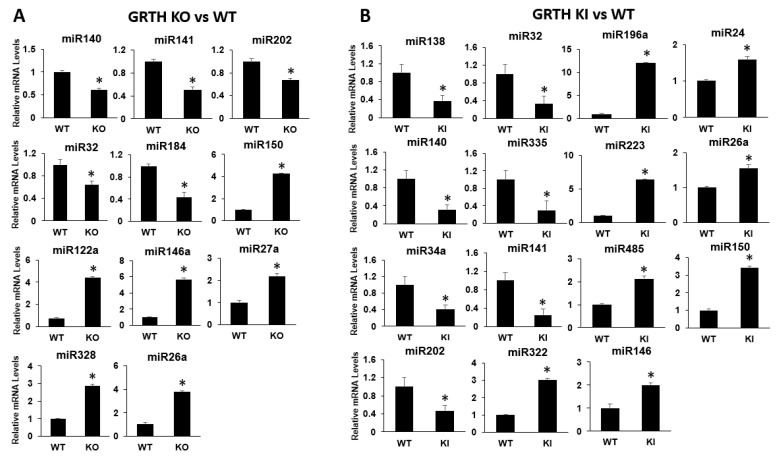
qRT-PCR analysis of selected miRNAs from RS of KO and KI groups. Bar graphs represent the fold change expression between (**A**) KO vs. WT or (**B**) KI vs. WT groups. Means ± SEM were determined from three independent qRT-PCR experiments with each sample run in triplicates using LNA probes. *p* values were calculated by two-tailed Student t-test (asterisks indicate *p* < 0.05).

Expression analysis of selected miRNAs from RS of KO and KI groups were compared with RS of WT mice using qRT-PCR analysis. Bar graphs represent the fold change expression between KO vs WT or KI vs WT group (Figure 5A,B). The miRNAs such as miR140, miR141, miR32, miR184, and miR202 show a decrease in abundance and miRNAs miR150, miR146, miR122a, miR27a, miR328, and miR26a show an increase in abundance in the RS of the KO mice (Figure 5A). In KI, miR138, miR140, miR34a, miR202, miR32, miR335, and miR141 show a decrease in abundance and miR196a, miR223, miR485, miR322, miR24, miR26a, miR150 and miR146 show an increase in abundance in the RS of the KI mice (Figure 5B). The results of qPCR studies correspond to expression profiles from the miRNA-seq data. The miRNAs, miR322, and miR24 target *Rnf138*, miR322 target *Pim1*, miR26a target *Ube2k*, *Jag1*, *Mical3*, *Pim1*, *Prkcq,* and *Hipk1* (Supplementary Table S5). All miRNA qRT-PCR experiments used LNA probes to obtain highly efficient expression data and validation.

### 3.6. Correlation of mRNA–miRNA Interaction to Understand Network of Interactions

**Figure 6:**
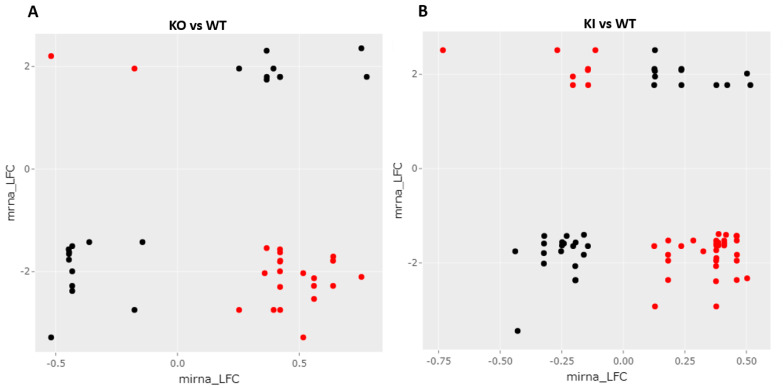
Correlation of mRNA–miRNA changes in the RS of KO, KI, and WT groups. mRNAs and miRNAs identified from the RS of (**A**) KO and WT group or (**B**) KI and WT group showing canonical correlation (Red, mRNA down and miRNA up or mRNA up and miRNA down) or non-canonical correlation (Black, mRNA down and miRNA down or mRNA up and miRNA up).

We selected the DEGs from mRNA-seq and miRNA-seq and then compared log2 fold changes between miRNA and putative mRNA targets based on the intersection of three published databases. The expected interaction relationship between a miRNA–mRNA pair should exhibit a negative correlation of expression (i.e., as miRNA expression increases, the expression of its mRNA target is expected to decrease). The mRNAs and miRNAs identified from the RS of KO, KI, and WT groups showing expected correlation (canonical, mRNA down = miRNA up or mRNA up = miRNA down) or not were highlighted in different color dots (Figure 6). Several target pairs are verified in this study to reveal the importance of these targets during late stages of spermiogenesis.

### 3.7. mRNA–miRNA Interaction Network Reveals Canonical mRNA–miRNA Role in Ubiquitination and Chromatin Compaction

**Figure 7:**
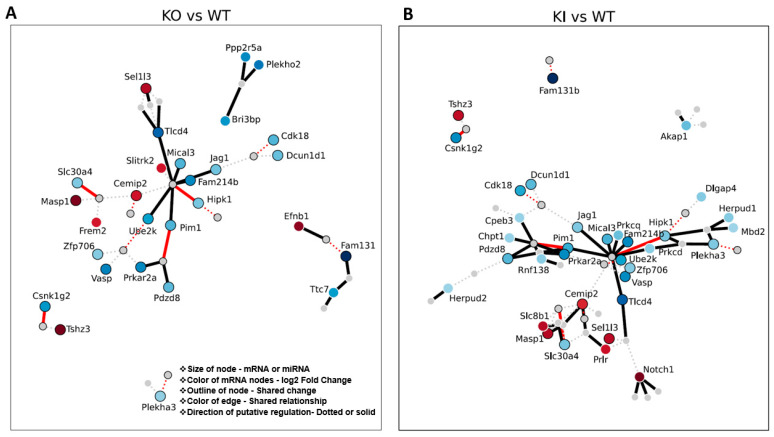
Putative mRNA–miRNA target regulation (from miRNA and mRNA DEGs) in the RS of KO, KI, and WT groups. mRNA–miRNA pairs in (**A**) KO vs. WT or (**B**) KI vs. WT group showing interaction (network). mRNA–miRNA pairs (only miRNA and mRNA changed in RS) in the genotype of interest showing interaction (positive or negative) identified jointly across databases. Red circles = upregulated compared to WT. Blue circles = downregulated compared to WT. Darker color circles = higher magnitude change. Each line represents a connection of a miRNA to an mRNA if it was identified jointly across databases. Thicker line = canonical change “miRNA down and mRNA up or miRNA up and mRNA down”. Dotted line = non-canonical “miRNA down and mRNA down or miRNA up and mRNA down”. Bright red line (dotted or not) = KO and KI behave similarly. Black outline around a node (circle) = node was shared in both KO and KI DEGs. A white outline around a node means that the node was unique to that genotype.

All mRNA–miRNA pairs showing both canonical or expected (miRNA down and mRNA up or vice versa) and non-canonical (miRNA down and mRNA down) interactions were used for the network construction. We observe essential protein-coding genes such as *Ube2k*, *RNF138*, *Pim1*, *Hipk1*, *Slc30a4*, and *Csnk1g2* regulated by miR26a (solid red lines indicate canonical for both KI and KO; solid black lines indicate just in KO or KI only) and other miRNAs mediate several essential pathways and intracellular function during spermatid differentiation and development. miRNAs (from the canonical mRNA–miRNA pairs) target multiple mRNAs and degrade them both in the cytoplasm and the CBs. The loss of mRNAs possibly resulted in the loss of essential proteins involved in ubiquitination, acrosome stability, and chromatin compaction that are required for spermatid differentiation and elongation events (Figure 7). The failure of these events resulted in spermatogenic halt and apoptosis of the RS.

### 3.8. mRNA–miRNA Interaction: miR122a Regulates Tnp2 Expression by Binding to Its 3′UTR

**Figure 8:**
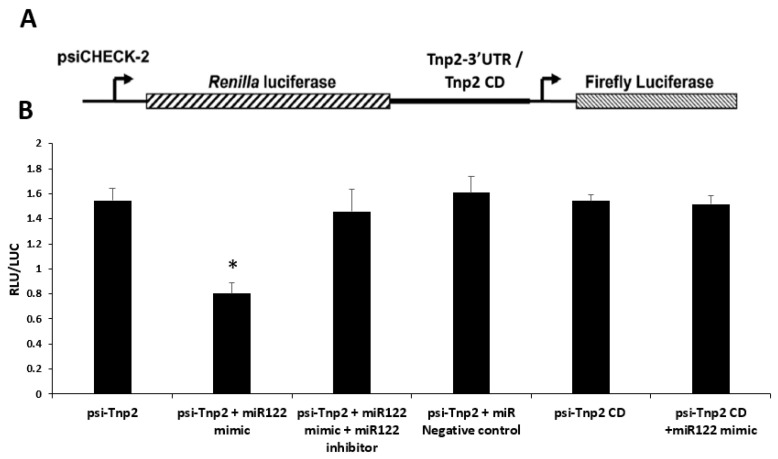
*Tnp2* mRNA–miRNA122a interaction. Modulation of translation of *Tnp2* depends on the presence of *Tnp2* 3′ UTR region. (**A**) Schematic representation of the psiCheck2 reporter gene carrying the *Tnp2* coding sequence with or without 3′ UTR. (**B**) Relative luciferase activity in COS-1 cells co-transfected with *Tnp2* with 3′UTR reporter constructs (or *Tnp2* coding region without 3′UTR) and miR122a mimic and/or miR122a inhibitor or negative control. Asterisks (*) indicate statistically significant change between psi-*Tnp2* and psi-*Tnp2* + miR122a mimic group (Student’s t-test; *p* < 0.05). All the data represent means ± SEM from three independent experiments, with each sample running in triplicates.

Temporal *Tnp2* gene translation is critical for the spermatid chromatin compaction process, which precedes protamine transition. Even subtle changes will impact the downstream processes in spermatid development that result in infertility [7]. miR122a was found to be upregulated in the RS of KO mice. Modulation of translation of *Tnp2* depends on the presence of regulators, such as specific miRNA, which binds to its 3′ UTR region and regulates it. To validate the interaction, a psiCheck2 luciferase reporter construct carrying the *Tnp2* coding sequence with or without 3′ UTR was used to transfect COS-1 cells. In addition, miR122 mimic or miR122 mimic together with miR122 inhibitor or miR negative control was used for co-transfection. A schematic representation of the psiCheck2 reporter gene carrying the *Tnp2* coding sequence with or without 3′ UTR is given in Figure 8A. The luciferase activity of *Tnp2* with 3′ UTR was decreased significantly in the presence of miR122a mimic. In contrast, *Tnp2* coding region (without 3′ UTR) did not alter the luciferase activity in the presence of miR122 mimic. To check the specificity of miR122a binding to the *Tnp2* 3′UTR, miR122a inhibitor was used together with miR122a mimic, which did not alter the luciferase activity (Figure 8B). This clearly shows that the miR122a binds to *Tnp2* at its 3′UTR in a sequence-dependent manner and decreases its translation specifically, which altered the TP2 protein levels that directly impacted later stages of spermiogenesis.

## 4. Discussion

Spermatogenesis is a highly controlled serial developmental process resulting in the formation of functional spermatozoa. During spermiogenesis, RS undergoes 16 steps of development with elongating, condensing, and condensed spermatids. Regulation of spermatogenesis occurs at multiple levels, starting from post-transcriptional to translational regulation. Previously, we have shown that the KO and KI male homozygous mice are infertile due to the arrest of RS at step 8 of spermiogenesis resulting in the loss of ES and mature sperm [5,7]. Single-cell RNA-seq analysis of testicular germ cells revealed a crucial role of GRTH in round spermatid differentiation into elongated spermatids and acrosome biogenesis [8]. Given the intricacies of different germ cell heterogeneity and specific differentiation pathways, GRTH/DDX25 plays a critical role in mRNA regulation both directly and by regulating miRNA biogenesis [11]. In this study, we used mRNA-seq, miRNA-seq data, and mRNA–miRNA interaction studies to address the changing gene expression signatures and the role of miRNA regulation in the developing RS. This study provides clues on the role of pGRTH and mRNA–miRNA dynamics in the developing RS of WT, KO, and KI mice.

In the current study, downregulation of several DEGs related to spermatogenesis, spermatid development, sperm motility, chromatin condensation, and DNA compaction were identified. Specifically, RNF138, UBE2K related to ubiquitination pathway and chromatin remodeling and transition proteins, *Tnp1/2*, *Prm1/2/3*, *Spata 3/18*, *Tssk3/6,* and several protein kinases and phosphatases altered during spermiogenesis. This current study demonstrated that the loss of pGRTH changed the transcriptomic profiles and may have indirectly impaired RNF138-, SPATA3-, and UBE2K-dependent histone modifications and nuclear transition protein-mediated chromatin remodeling [32]. This directly impacted the initiation of spermatid elongation at step 8 of spermiogenesis. Ring finger protein 138 (RNF138) is a member of an E3 ligase family that has been shown to be recruited to the regions of DNA double-strand breaks and repair them by homologous recombination [33]. Rnf138 is highly expressed in spermatogonia and spermatocytes, and Rnf138 deficiency promotes apoptosis of spermatogonia [34]. Histone ubiquitination and acetylation play a crucial role in chromatin remodeling, which is essential for the development of spermatids during spermiogenesis [35,36]. During step 8 of spermiogenesis, hyperacetylation of histones (H) leads to the replacement of histone followed by nuclear elongation and extension of the acrosome. Ubiquitin-conjugating enzyme E 2K interacts with Ring finger proteins, and its deficiency causes the failure of germ cells to undergo meiosis, resulting in male infertility. UBE2K is a component of the PRC1 complex that ubiquitinates histone H2A. UBE2K, in combination with RNF138, robustly induces the formation of ubiquitinated H3 [37]. The early-stage germ cells synthesize transcripts of these proteins and store them prior to nuclear condensation events. Differential expression of important mRNAs that mediate these events resulted in spermiogenesis halt.

The final stages of spermiogenesis require several proteins that are essential for the differentiation of the RS, such as proteins involved in chromatin remodeling and compaction processes. During the chromatin remodeling process, 90% of the nucleosomal histones are replaced by testis-specific TP1/2. Subsequently it is replaced by sperm-specific PRM1/2 to form a highly condensed spermatid/sperm chromatin [38,39]. Differential expression analysis identified several genes such as *Tnp1/2*, *Prm1/2/3*, *Spem1/2*, *Spata3/18*, and *Tssk3/6*, which play critical roles in spermatid development, elongation, and chromatin compaction that were downregulated in KI and KO mice. SPATA 3 (spermatogenesis-associated protein 3), also known as Tsarg1, is expressed specifically at lower levels in pachytene spermatocytes and peaks in spermatids [40,41]. SPATA3 interacts with KLHL10, which is expressed exclusively in spermatids, and its inactivation leads to the disruption of spermiogenesis and complete male infertility in mice [42]. KLHL10 is a substrate-specific adapter that interacts with CUL3 (Cullin3), a core component of cullin-RING-based E3 ubiquitin-protein ligase complex functions specifically during spermiogenesis [43]. Our data shed light on the plausible role of SPATA3 and the involvement of GRTH in the SPATA3-, UBE2K-, and KLHL10-mediated protein ubiquitination pathway during spermiogenesis. The current study also identified mRNAs of several protein kinases and phosphatases, such as *Csnk1g2*, *Hipk1*, *Prkcq*, *Pim1*, and *Ppp2r5a*, which mediate several intercellular protein regulations inside developing spermatids which were targeted by miRNAs. Transition proteins and protamines are arginine-rich nuclear proteins that replace histones late in the haploid phase of spermatogenesis. Lack of these proteins leads to impaired nuclear condensation in the spermatid head, resulting in infertility. GRTH binding of *Tnp2* mRNA decreased significantly in KI mice testis and inside the CB of germ cells [4]. Increased miR122a levels in the loss of GRTH in KO mice suggest that GRTH might have an intrinsic regulatory role in regulating the levels of miR122a. Another important miRNA identified in this study is miR26a, which targets several important mRNAs such as *Ube2k*, *Rnf138*, *Pim1*, *Hipk1*, *Slc30a4*, *and Csnk1g2.* These genes mediate several essential pathways and intracellular functions, which are critical for spermatid differentiation and development. miR26a regulates *Jag1* (NOTCH ligand) expression, thereby regulating GDNF expression in Sertoli cells [44]. It also regulates sperm apoptosis by directly targeting PTEN and has a link with decreased sperm motility [45]. There is a significant overexpression of miR27a in infertile men with nonobstructive azoospermia [46], which target several genes, including H3K9 demethylase that regulates transcriptional suppression of *Tnp1/Prm1*, resulting in infertility in animal models and humans [47,48]. *Prkar2a* codes for cAMP-dependent protein kinase type II-alpha regulatory subunit (enzyme) and is regulated by miR322 in the RS of KO and KI mice. Prkar2a is involved in cAMP signaling and mediates membrane association by binding to the anchoring proteins, including the microtubule-associated protein 2 (MAP2) kinase. A-kinase anchoring proteins are functionally diverse polypeptides that compartmentalize PKA within the cell and are critical due to their unique ability to directly and/or indirectly interact with proteins that determine the cellular content of cAMP. The study identified miR130b as the targeting miRNA which acts on *akap1* and decreases its expression. AKAP1 is a transcriptional target of Myc and supports the growth of cancer cells [49]. These are strong interactions and correlations between several kinases and phosphatases, and spermatid development.

Transgenic mice studies showed that the 3′ UTR of *Prm1/2* and *Tnp1/2* is responsible for the correct temporal translation of their mRNAs during spermatogenesis. Putative functional response elements were identified within the coding region of Tp2 and Prm2 [11], and here we confirmed that miR122a targets *Tnp2* by binding to 3UTR. Although there was no significant differential expression of miR122a in our miRNA-seq data, we found a significant reduction (4-fold) in their levels in qPCR expression analysis. This discrepancy between assays may be due to the fact that high-throughput sequencing libraries, by necessity, use the same number of PCR cycles for all transcripts, which may not be optimal for low-expression transcripts such as miR122a. In addition, luciferase reporter assays validated the interaction of miR122a and *Tnp2* mRNA. miR122a binds to 3′ UTR of *Tnp2* and modulates its translation in a site-specific manner which directly impacts later stages of the spermiogenesis process. miR122-5p was shown as a potential biomarker of male infertility [17]. Translation of *Prm2* and *Tnp2* mRNA were destined for ES stages (from steps 9–16 of spermiogenesis) but were actively transcribed early in the RS stage and stored temporarily inside the CB [4]. High levels of miR122a present during the RS stage result in their binding to *Tnp2* mRNA and subsequently suppressing its translation. Furthermore, GRTH is one of the essential components of the CB, which also harbors miRNA–mRNA control mechanisms that mediate post-transcriptional regulation, including mRNA silencing or processing during spermiogenesis [9]. These findings could provide novel insights into the role of small RNAs and their interaction with a selective group of important mRNAs at the post-transcriptional level during spermiogenesis.

Taken together, our studies indicate that the identified miRNAs target several mRNAs involved in ubiquitination, histone removal, and chromatin compaction processes, thereby controlling post-transcriptional regulation resulting in spermatogenic halt in KO and KI mice. In conclusion, pGRTH plays a critical role during RS development through miRNA-mediated mRNA regulation, thereby maintaining the overall regulation at the transcriptional, post-transcriptional, and translation levels.

## Figures and Tables

**Table 1:** Important genes and their associated functions.

Rnf138	Ring Finger Protein 138, E3 ubiquitin-protein ligase involved in DNA damage response, highly expressed in spermatogonia and spermatocytes. Rnf138 deficiency promotes apoptosis of spermatogonia in male mice
Ube2k	Ubiquitin conjugating enzyme E2 K, interact with Ring finger proteins, Ube2k deficiency cause failure of germ cells to undergo meiosis results in male infertility
Csnk1g2	Casein Kinase 1 Gamma 2, enables protein serine/threonine kinase activity, involved in peptidyl-serine phosphorylation, CSNK1G2-attenuated necroptosis mediate testis-aging program, CSNK1G2-knockout mice showed significantly enhanced necroptosis response and premature aging of testis
Hipk1	Homeodomain-interacting protein kinase 1, phosphorylates homeodomain transcription factors (acts as co-repressor)
Pim1	Encodes for the serine/threonine kinase, role in regulation of DNA damage repair
Jag1	NOTCH ligand, JAG1 regulates GDNF expression in Sertoli cells
Mical3	Microtubule-Associated Monooxygenase, Calponin, and LIM Domain Containing 3, MICAL3 work together in the process of docking and fusing of vesicles that are involved in exocytosis
Pdzd8	PDZ domain containing 8, cytoskeletal regulatory protein that interacts with moesin and regulates stable microtubule abundance, acrosome stability.
Ppp2r5a	Major Ser/Thr phosphatases, role in negative control of cell growth and division
Akap1	A-kinase anchoring protein, role in Star production and steroidogenesis
Mbd2	Methyl-CpG Binding Domain Protein 2,
Prkcq	Protein kinase C theta, member of serine/threonine kinases, critically regulates cell growth and survival, mutation leads to male infertility

## Data Availability

The mRNA-Seq and miRNA-seq data files (KO, KI, and WT samples) in this study have been submitted to the NCBI (https://www.ncbi.nlm.nih.gov/geo, accessed on 22 February 2023) Gene Expression Omnibus (GEO accession number: GSE222628; Super Series record). All other RNA-Seq results analyzed during this study are included in this article and its supplementary files.

## Supplementary Materials

The following supporting information can be downloaded at: www.mdpi.com/article/10.3390/cells12050756/s1. Figure S1: PCA plot of mRNA samples colored by genotype. Note KI sample is nearly hidden in the lower right. Supplementary Figure S2: PCA plot of miRNA samples colored by genotype. Note the strong batch effect (PC1) that was controlled for during differential expression analysis. Supplementary Figure S3: Combinatorial overlaps of mRNAs A. Downregulated or B. Upregulated in KO, KI, and WT groups. Supplementary Figure S4: Combinatorial overlaps of miRNAs A. Downregulated or B. Upregulated in KO, KI and WT groups. Supplementary Table S1: List of primers used for validation of mRNA-seq using qPCR. Supplementary Table S2: List of LNA probes used for validation of miRNA-seq using qPCR. Supplementary Table S3: List of DEGs, mRNA from KO, KI, and WT mice RS. Supplementary Table S4: List of DEGs, miRNA from KO, KI, and WT mice RS. Supplementary Table S5: Canonical mRNA-miRNA interaction pairs (all) from the DEGs in the KI and KO mice RS.
