# Supplementary material for: miRNA Expression Profiles of Mouse Round Spermatids in GRTH/DDX25-Mediated Spermiogenesis: mRNA–miRNA Network Analysis
Source: Cells. 2023 Feb 27;12(5):756. doi: 10.3390/cells12050756 (PMC10001410; doi:10.3390/cells12050756)
Supplement: Supplementary file 1 [file cells-12-00756-s001.zip › Supplementary Table S1.pdf]

**Primer Name**	**Primer Sequence 5'---3'**
Rnf138 F	GAG GAT ACA AGT TCT TCT GGG CA
Rnf138 R	GGA ACT ATC TGA AAT AGG TGG TTA C
Ube2k F	GTA GCG AAT CAG TAC AAA CAG AAT CCT
Ube2k R	GAC TAG AAA CTG GTG CTC CAG C
Csnk1g2 F	GCT CAA CTC CAC TAA TGG AGA GCT
Csnk1g2 R	GCA GCA GCA CTT TGT TTC ATC TGC
Hipk1 F	GAC CTG CAG CAG ATG GCA TTG
Hipk1 R	GCT AAG GAG ACC TGA GGC CTG
Pim1 F	GTC TCT TCA GAG TGT CAG CAC C
Pim1 R	GCA TCC ATG GAT GGT TCC GGA T
Jag1 F	GTG GCC ATC TCT GCA GAA GAC A
Jag1 R	GCT GTT TCC ATC CCG TTT ACT AAC G
Mbd2 F	GAT GTC TAC TAC TTC AGT CCA AGT GG
Mbd2 R	GCA TTT CCC AGG TAT CTT GCC AG
Mical3 F	GCT GAT GAT TTT TGC CCG GGA GC
Mical3 R	GTC TTC AGG TGA TCT TCC ACG GCC A
Prkcq F	GAG ATC AAC TGG GAA GAG CTT G
Prkcq R	GGT GAT TTC ACT TTT GGT CTG AAG G
Akap1 F	GCA GAC GCA GCC ATG AGT GAG
Akap1 R	GTA GCT TGT CAC CTG GGC CA
Ppp2r5a F	GAG CAC TGT ACT TCT GGA ATA ATG
Ppp2r5a R	GTG CTA CAA TAG TCT GAT TCC AGT G
Pdzd8 F	GCT ATT GGA GGT GTG AAA ATC ACA
Pdzd8 R	GTA GTA CAC CAG CAC GCG GT
18SrRNA F	GCA ATT ATT CCC CAT GAA CG
18SrRNA R	GGC CTC ACT AAA CCA TCC AA
