# Supplementary material for: miRNA Expression Profiles of Mouse Round Spermatids in GRTH/DDX25-Mediated Spermiogenesis: mRNA–miRNA Network Analysis
Source: Cells. 2023 Feb 27;12(5):756. doi: 10.3390/cells12050756 (PMC10001410; doi:10.3390/cells12050756)
Supplement: Supplementary file 1 [file cells-12-00756-s001.zip › Supplementary Table S2.pdf]

miR Name	miRCURY LNA miRNA Probes (PCR Assay Number)
miR140-5p	YP00204540
miR141-5p	YP00205321
miR202-5p	YP00205654
miR150-5p	YCP2141991
miR146a-5p	YCP2141994
miR122a-5p	YCP2141997
miR27a-5p	YCP2142000
miR328-5p	YP02116348
miR26a-5p	YP00206023
miR138-5p	YP00206078
miR184-5p	YP00204601
miR32-5p	YP00204792
miR196a-5p	YP00204386
miR24-3p	YP00204260
miR335-5p	YP02119293
miR223-3p	YP00205986
miR34a-5p	YP00204486
miR485-5p	YP02112548
miR322-5p	YP00205182
U6 snRNA	YP00203907
