# Supplementary figures and images for: miRNA Expression Profiles of Mouse Round Spermatids in GRTH/DDX25-Mediated Spermiogenesis: mRNA–miRNA Network Analysis
Source: Cells. 2023 Feb 27;12(5):756. doi: 10.3390/cells12050756 (PMC10001410; doi:10.3390/cells12050756)

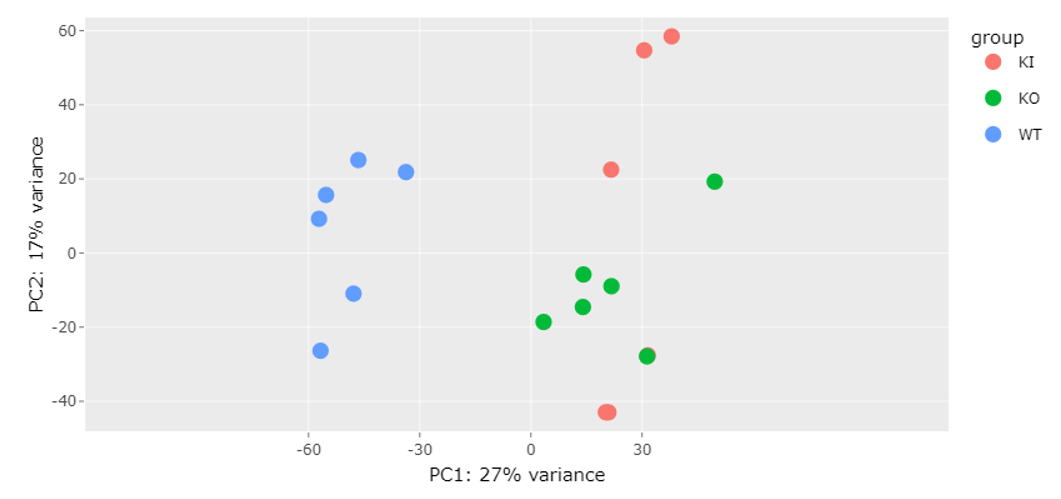

Supplement: Supplementary file 1 [file cells-12-00756-s001.zip › Supplementary figure S1.png]

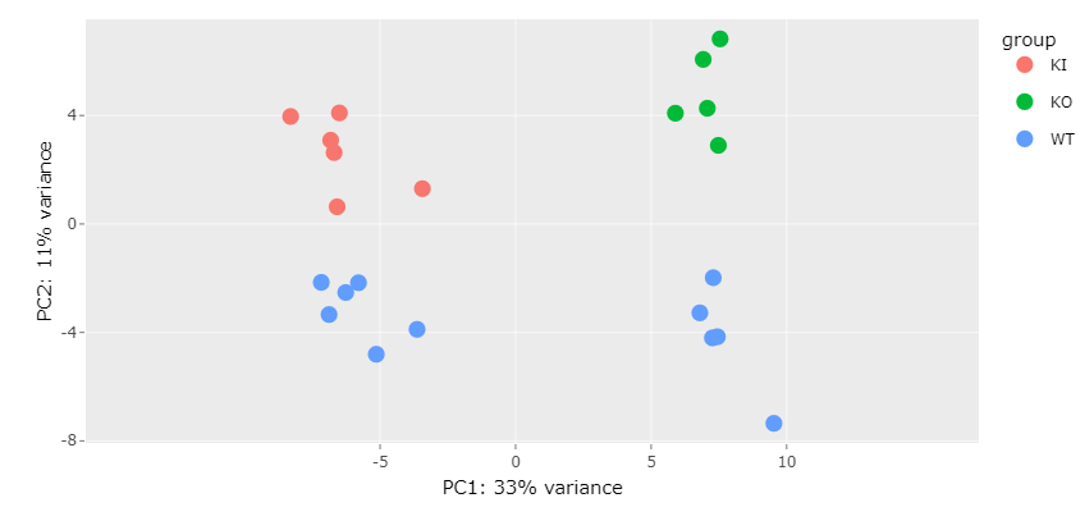

Supplement: Supplementary file 1 [file cells-12-00756-s001.zip › Supplementary figure S2.png]

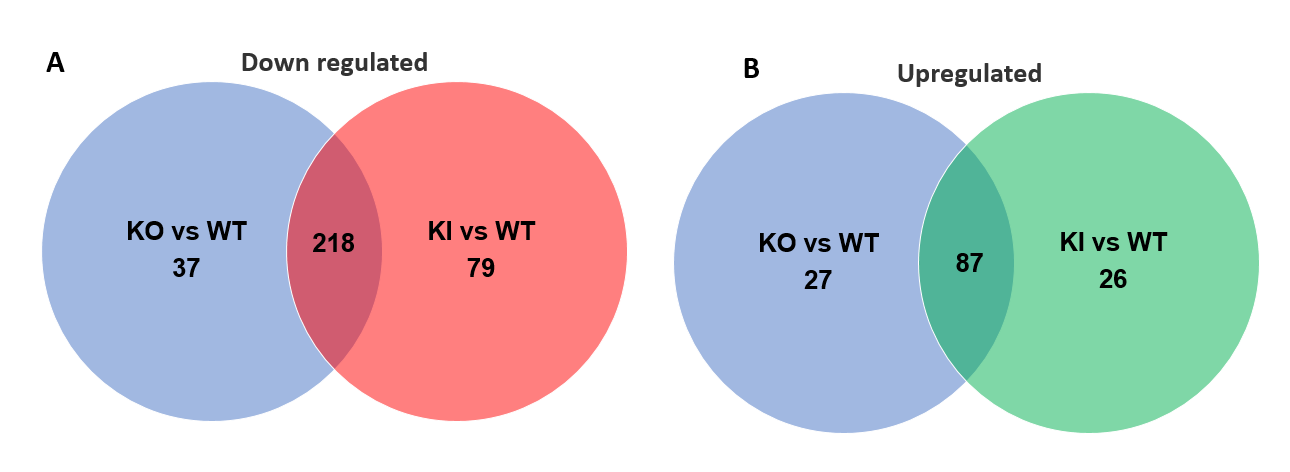

Supplement: Supplementary file 1 [file cells-12-00756-s001.zip › Supplementary figure S3.png]

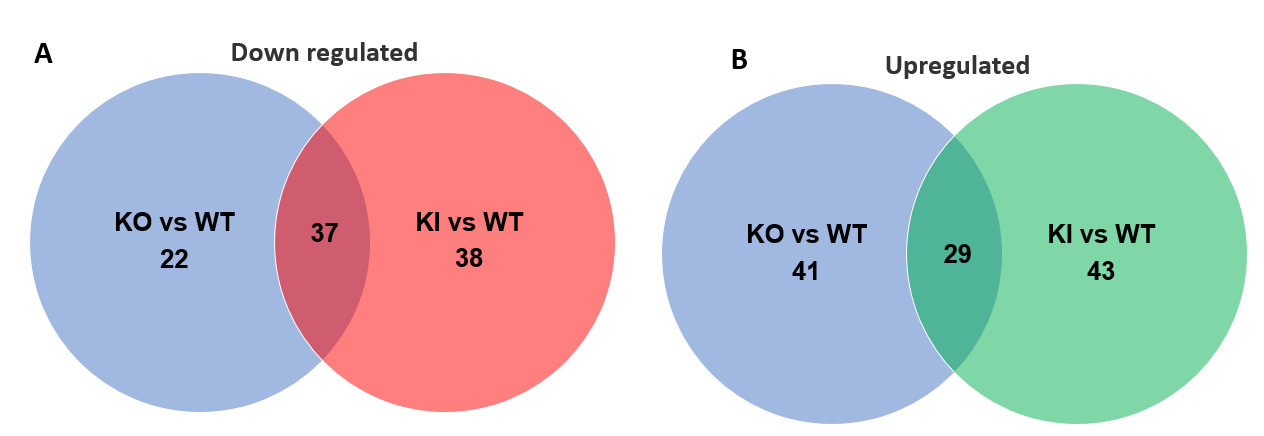

Supplement: Supplementary file 1 [file cells-12-00756-s001.zip › Supplementary figure S4.png]
